# Genomic Insights Into the Distribution and Evolution of Group B Streptococcus

**DOI:** 10.3389/fmicb.2019.01447

**Published:** 2019-06-28

**Authors:** Swaine L. Chen

**Affiliations:** ^1^Division of Infectious Diseases, Department of Medicine, Yong Loo Lin School of Medicine, National University of Singapore, Singapore, Singapore; ^2^Infectious Diseases Group, Genome Institute of Singapore, Singapore, Singapore

**Keywords:** group B Streptococcus, *Streptococcus agalactiae*, foodborne disease, review, genomics, molecular epidemiology, whole genome sequencing

## Abstract

*Streptococcus agalactiae*, also known as Group B Streptococcus (GBS), is a bacteria with truly protean biology. It infects a variety of hosts, among which the most commonly studied are humans, cattle, and fish. GBS holds a singular position in the history of bacterial genomics, as it was the substrate used to describe one of the first major conceptual advances of comparative genomics, the idea of the pan-genome. In this review, I describe a brief history of GBS and the major contributions of genomics to understanding its genome plasticity and evolution as well as its molecular epidemiology, focusing on the three hosts mentioned above. I also discuss one of the major recent paradigm shifts in our understanding of GBS evolution and disease burden: foodborne GBS can cause invasive infections in humans.

## Introduction

*Streptococcus agalactiae* was first described in 1887 as a common bacteria infecting the udders of cattle, causing a disease termed mastitis (Nocard and Mollereau, 1887). This led to significantly lower milk production (Keefe, 1997; Ruegg, 2017), thus the species name *agalactiae* (from the Greek: a-, no; galactos, milk). In these cows, milk production can be reduced over 20% in infected cows, which, prior to the institution of active control measures, could conservatively affect 15–40% of all cows (Shaw and Beam, 1935). It came to be known by its other common name, Group B Streptococcus, with the Lancefield classification in 1933 (Lancefield, 1933), where the “Group B” references the species-specific carbohydrate “substance C” from Streptococci (Lancefield, 1928) recognized by rabbit immune sera (this substance C is associated with the cell wall and distinct from the polysaccharide capsule). Today, GBS is well known as the most common cause of neonatal meningitis, which is further classified into early onset (<7 days after birth) and late onset (7 days to 3 months after birth) (Berardi et al., 2007; Edwards et al., 2011; Nanduri et al., 2019). Transmission to the newborn can be vertical, through contact with mucus membranes, or through ingestion of infected amniotic fluid (Verani et al., 2010). Consistent with this, it is a frequent (20–40% of individuals) colonizer of the human gastrointestinal tract and the reproductive tract of women, based on two prevalence studies in a North American university (Bliss et al., 2002; Manning et al., 2004). However, GBS is also an increasingly common cause of severe invasive disease, typically in immunocompromised individuals and the elderly, since the 1960s (Farley et al., 1993; Schuchat, 1998; Farley, 2001; Francois Watkins et al., 2019).

These well-known facts about the history and medical importance of GBS parallel several deeper themes of GBS biology. The dual names by which we refer to this bacterium echo a split in how we have studied its biology in humans, cattle, and other animals. The rise of human infections, first in neonates and more recently in adults, matches a theme of ongoing evolution and niche expansion for the species. Finally, the shift in associated species from cattle to humans foreshadows additional potential species jumps that are apparently continuing to this day. GBS is now known to be widely distributed among diverse species of mammals, reptiles, amphibians, and fish (such as dogs, cats, goats, elephants, frogs, crocodiles, dolphins, seals, llamas, and camels) (Edelstein and Pegram, 1974; Bishop et al., 2007; Delannoy et al., 2013; Tavella et al., 2018), not only colonizing but in many cases also capable of causing severe invasive disease. Of particular note, besides its importance in human and bovine medicine, GBS is a significant pathogen for aquatic species, including those of importance for food production (Amal et al., 2011). Streptococcal infections are responsible for an estimated US$150 million in global losses in farmed tilapia in 2000 (close to 10% of the total value) (Amal et al., 2011). As with human and bovine infections, GBS infection in fish was first described relatively recently, with two seminal reports in 1958 and 1966 (Hoshina et al., 1958; Robinson and Meyer, 1966). Even from these first reports, GBS was noted to be a particular danger to farmed fish, being highly contagious and usually fatal (Hoshina et al., 1958; Robinson and Meyer, 1966).

GBS has therefore been studied in multiple contexts: human health, veterinary medicine, and agriculture. Research has thus been motivated by both health and economic goals, which naturally vary in importance across these different disciplines. Beyond the proximal questions of how GBS colonizes and causes specific diseases in specific hosts, GBS is an intriguing case study for the larger questions of how broad host range at the species level is maintained despite evidence of variation at the subspecies level. These larger evolutionary questions are again made more urgent by convincing evidence of ongoing adaptation and emergence of pathogenicity and resistance in GBS.

To tackle these specific questions about disease mechanisms as well as broader evolutionary questions, genomics is a natural fit. Recent years have seen an explosion of genomic data available for GBS, as is the case for all other bacteria. The transition to a post-genomic era for GBS holds an additional promise for a unified understanding across the medical and veterinary fields, which may lead to a fuller appreciation of the importance and impact of this versatile bacterium. In this review, I will focus on two primary topics: (1) GBS genome plasticity and evolution and (2) GBS molecular epidemiology related to geography and host range (focused on humans, cattle, and fish). I have included some contextual information drawn from non-genomic papers using other typing systems, but this review does not aspire to be complete in regard to the entire corpus of GBS studies.

## Pre-Genomic Classification Systems

GBS, like many other bacteria of medical importance, has long been recognized to have intraspecies variation that can be tracked with a variety of molecular methods. Early studies utilized immunological reactions, resulting in a (still commonly used) serotyping scheme consisting of 10 major serotypes (Ia, Ib, II-IX) (Edwards et al., 2011). Numerous other systems have been applied to GBS, including ribotyping (Huet et al., 1993), RAPD (Random Amplification of Polymorphic DNA) (Limansky et al., 1998), RFLP (Restriction Fragment Length Polymorphism) (Hauge et al., 1996), PFGE (Pulsed-Field Gel Electrophoresis) (Rolland et al., 1999), and MLEE (Multilocus Enzyme Electrophoresis) (Musser et al., 1989). The other major pre-genomic classification system (although, ironically, published after the first genome sequences became available) that still remains in common use today is MLST (Multilocus Sequence Typing) (Jones et al., 2003), due to its balance between ease of typing, portability between labs, and reasonable resolution (Maiden, 2006). From these initial pre-genomic studies, the general outlines of the population structure of GBS were inferred. The different major hosts (humans, cattle, and fish) have largely distinct populations of strains, with some notable exceptions that may be indicative of cross-species jumps. Furthermore, changes in the epidemiology of disease and responsible serotypes or MLST types have been noted (see Section “The Pan-Genome”). However, overall, prior to the genomic era, GBS could generally (though not exclusively) be classified based on its host species (Finch and Martin, 1984; Bohnsack et al., 2004; Sukhnanand et al., 2005; Evans et al., 2008; Pereira et al., 2010), which then could be subdivided into several (3–5) large groups of closely related strains (termed clonal complexes in the MLST nomenclature) that accounted for most diseases (Table 1).

**Table 1:** Major MLST Clonal Complexes of GBS found in humans, cattle, and fish.

MLST clonal complex	Associated serotype(s)	Associated host(s)	Associated geography
1	III, V, VI, VIII	Human	Global
1 (ST459)	IV	Human	North America
7	Ia	Fish, Human	Asia (fish)
10	Ib	Human	Global
10 (ST283)	III	Human, Fish	Southeast Asia, Brazil
17	II, III	Human, Cattle	Global
19	II, III	Human	Global
23	Ia, III	Human, Cattle	Global
26	IV	Human	Africa
67	Ia, II	Cattle	Americas, Europe
103	Ia	Cattle	Europe, Asia
552	Ib	Fish	Global

## Group B Streptococcus Genome Plasticity and Evolution

### Initial Genome Sequences

The first full genome sequences of GBS were of the human isolates NEM316 (ST23, Serotype III) (Glaser et al., 2002) and 2603V/R (ST110 (a single locus variant of ST19), Serotype V) (Tettelin et al., 2002), both published in late 2002. These two genomes provided a heretofore unprecedented view of the organization and potential evolution of the GBS genome, which was initially gleaned from comparisons with genomes of *S. pyogenes* and *S. pneumoniae* strains. Generally, the GBS genome consists of a conserved “backbone” that is punctuated by 14–15 genomic islands of variable gene content (and many smaller islets). GBS was remarkable for its high number of tRNAs (80), ABC transporters (62), and signal transduction systems (17–20 two component systems). In addition, there are multiple classes of mobile DNA elements that presumably contributed to disrupting, duplicating, and transferring genes. In particular, insertion sequences, prophages, and a triplicated integrated plasmid in NEM316 (denoted pNEM316–1) (Glaser et al., 2002) were major contributors to variation in gene content that was specific to GBS and that also varies among different GBS strains. With only a single full genome to analyze, neither of these initial genome papers were able to directly identify large-scale chromosomal recombination occurring in GBS, though it was clear from comparative genome hybridization experiments that genes within the species-specific GBS islands were also more likely to vary among GBS strains as well (Tettelin et al., 2002).

### The Pan-Genome

The structure of the GBS genomes, with a conserved backbone punctuated with islands of variable gene content, led directly to a simple hypothesis as to why different GBS strains may preferentially colonize or infect different hosts. GBS as a species might be defined by the conserved regions, while the variable islands, which often possessed features of genes important for virulence (Glaser et al., 2002; Tettelin et al., 2002), could carry genes that provided specific phenotypes important in different hosts or different disease settings. This structural stratification of gene conservation became more clear as more genomes were sequenced, not just for GBS but for other bacteria as well, most notably *E. coli* (Welch et al., 2002).

This represented, in a sense, a specific genomic extension to genetics. Clearly, different phenotypes for host specificity and disease would be traceable to genetic mechanisms; and now, perhaps, there was a structural genomic framework which would organize the adaptation and evolution of these traits. The first formalization of this idea was the pan-genome concept (though the potential *structural* aspect of genome organization was not noted). The seminal paper describing the concept of a pan-genome (the complete set of genes that is found in all individuals of a given species) was based on an analysis of eight GBS genome sequences (Tettelin et al., 2005). GBS therefore holds a special place in the early transition to the post-genomic era for bacteria. It was also the first organism described to have an “open” pan-genome; rarefaction analysis predicted that, even with an arbitrarily large number of genome sequences, every new genome sequence would contribute an extra 33 genes that had not previously been seen in any other GBS. The pan-genome concept was one of the first truly new results to arise from comparative genomics; it was a systematic rationalization of differing gene content that necessarily required the existence of multiple genome sequences, and it further gave rise to the companion concept of a core genome that consists of genes that are conserved across all individuals of a given species (Medini et al., 2005; Tettelin et al., 2005).

The initial ideas about core and pan-genomes were a remarkably useful organizational framework for thinking about a variety of issues relevant to bacteria and genomics, leading to their immense popularity. The pan-genome concept was closely related to ideas about genome plasticity, horizontal gene transfer, the concept of species for bacteria, and genome evolution (Medini et al., 2005). The conceptual simplicity of core (conserved) versus accessory (variable) genes in an organism was a natural fit for rationalizing differences in pathogenic potential, host range, and other variable phenotypes. Put simply, a core genome in some sense defined a species by providing conserved phenotypes and responses; of prime practical interest were those that medical microbiology leveraged to perform species identification in the lab. For an individual strain, the accessory genome (in other words, the subset of the pan-genome found in that individual above and beyond the core genome) could vary from other individuals, and would explain differences in observed phenotypes such as virulence; alternatively, as more genomes became sequenced, it became clear that sometimes gene set differences could also be related to different ecological niches, such as different geographical locations or host organisms. It was also further shown that large (up to 334 kb) chromosomal segments, including these islands, could be transferred horizontally between strains (Brochet et al., 2008).

The initial promise of the pan-genome concept for providing a holistic organizational framework for individual bacterial species, however, was difficult to fulfill. The initial eight GBS strains that were sequenced and analyzed were chosen for their representation of different serotypes and host organisms, both proxies for sampling the diversity of the species and its disease-relevant phenotypes (Tettelin et al., 2005). One implicit assumption embedded in this initial analysis was that new GBS genomes would sample similarly new subsets of the species diversity; returning to serotype or host organisms as proxies, larger data sets would inevitably begin to sample (at least some) very similar strains. In other words, the initial analysis looked at eight strains that were first chosen for sequencing because they were of different serotypes and MLSTs; they were chosen to represent the diversity of the species. With hundreds to thousands of genomes, however, an additional strain is unlikely to represent a divergent, previously unsampled clade or subclone. Thus the diversity modeled from eight relatively diverse strains may not be accurate when extrapolated to an arbitrary number of strains. Indeed, the high relatedness of many GBS isolates has become more clear in the observation of frequent clonal expansion of virulent or hypervirulent clones, especially among those causing disease (see examples below in the “Molecular Epidemiology” sections).

In addition, the original pan-genome paper inspired numerous similar analyses on other sets of genome sequences, and not all were limited to gene sequences (Lefébure and Stanhope, 2007; Liu et al., 2013; Kayansamruaj et al., 2015; Puymège et al., 2015; He et al., 2017; Wolf et al., 2018; Wang et al., 2018a); the result of an open pan-genome was consistently found. Beyond the use of genomics to calculate the sizes and “openness” of the core and pan-genomes, many studies performed additional analyses that provided several clear and general insights into overall GBS genome plasticity and evolution. GBS genomes have obviously evolved by recombination, likely driven by large-scale DNA transfers mediated by mobile elements (Bröker and Spellerberg, 2004; Brochet et al., 2008; Sørensen et al., 2010; Da Cunha et al., 2014). There is an interesting dichotomy between very clear evidence of large-scale recombination between different lineages of GBS (Springman et al., 2009; Da Cunha et al., 2014; Teatero et al., 2016; Campisi et al., 2016a) with very little recombination within expanding clones, which instead evolve largely by accumulation of mutations (Brochet et al., 2006; Flores et al., 2015; Almeida et al., 2017; Kalimuddin et al., 2017). Interestingly, there have been several noted instances of serotype switching, likely through recombination and after emergence of a successful lineage, which may confound earlier typing studies (Luan et al., 2005; Brochet et al., 2008; Martins et al., 2010; Bellais et al., 2012; Teatero et al., 2014; Neemuchwala et al., 2016; Wang et al., 2018a). These can occur through large-scale recombinations (most clearly seen in originally Serotype V ST1 strains that have converted to Serotype Ib, II, and IV through apparently single recombinations, ranging from 79 to 200Kbp, encompassing the capsule determining *cps* locus) (Neemuchwala et al., 2016). Notably, genomics provides the most clear view of this phenomenon, as previous studies using PFGE, MLST, lab-based serotyping, and sequencing of the cps locus estimated potential serotype switching events from 2 to 16% (Luan et al., 2005; Martins et al., 2010).

Therefore, the overall picture of GBS evolution is consistent with the hypothesis, most clearly articulated by the lab of Philippe Glaser, of a continuous generation of new lineages, through any mutational mechanism including mobile element activity, reductive evolution, or large-scale recombination, coupled with nearly clonal evolution of successful lineages, characterized by very little recombination and possibly by reductive evolution (Brochet et al., 2006, 2008; Lefébure and Stanhope, 2007; Sørensen et al., 2010; Rosinski-Chupin et al., 2013; Almeida et al., 2016). There are several additional strong results that provide insights into the mechanisms of subspecies adaptation. Notable examples are the consistent genome reduction in fish-adapted isolates (see Section “Group B Streptococcus Molecular Epidemiology in Fish”) (Liu et al., 2013; Rosinski-Chupin et al., 2013; Delannoy et al., 2016); the consistent presence of the *scpA* and *lmb* virulence factors in human isolates (though they are still found, at lower frequency, in animal isolates) (Franken et al., 2001; Sørensen et al., 2010); and the development of metabolic modifications matched to expected nutrient sources, exemplified by the acquisition of the Lac.2 operon enabling lactose fermentation in cow-associated strains (Richards et al., 2013).

### Group B Streptococcus Genome Sequencing Today

As of this writing (March, 2019), there are over 7,000 GBS strains for which genome sequencing data are publicly available in the GenBank Sequence Read Archive (SRA). As with other microorganisms, the number of data sets has grown exponentially over the past few years, and the literature contains reports of several notable survey studies that together have contributed several thousand data sets (Metcalf et al., 2017), though several of these appear not to have been published in manuscripts yet (Table 2). Furthermore, the advent of journals like Genome Announcements, which publishes only genome sequences without analysis, has led to a large growth in manuscripts describing single or multiple genome sequences, and genome sequencing is being more routinely used as a tool (as opposed to the main endpoint of a study) (Table 3).

**Table 2:** Large BioProjects listed in GenBank with more than 100 Illumina whole genome sequencing data sets for GBS (As of March 7, 2019).

Project title	Data sets	Contributor	BioProject IDs	Reference
Invasive group B streptococcal isolates	4,381	Centers for Disease Control and Prevention	PRJNA355303 SRP094065	(Metcalf et al., 2017)
Group B streptococcal infections in neonates. GBS interaction with the host innate immune system	1,512	Wellcome Trust Sanger Institute	PRJEB14124 ERP015737	
The Clinical and Molecular Epidemiology of *Streptococcus agalactiae* (GBS) Colonisation on the Kenyan Coast	1,034	University of Oxford	PRJNA315969 SRP072745	(Seale et al., 2016)
*Streptococcus agalactiae* group B Streptococcus GBS associated with maternal carriage in the UK	743	Wellcome Trust Sanger Institute	PRJEB20117 ERP022239	
Comparison of molecular serotyping approaches of *Streptococcus agalactiae* from genomic sequences	800	Public Health England	PRJEB18093 ERP020015	(Kapatai et al., 2017; Jauneikaite et al., 2018)
Whole genome sequencing and analysis of *Streptococcus agalactiae* samples collected in Malawi	379	University of Oxford	PRJEB8986 ERP010039	
WGS of Group B Streptococcus from disease and healthy carriage	355	Wellcome Trust Sanger Institute	PRJEB11000 ERP012314	
Singapore *S. agalactiae* working group sequencing project	351	Genome Institute of Singapore	PRJNA293392 SRP078405	(Chau et al., 2017; Kalimuddin et al., 2017)
Genomic characterisation of uropathogenic S*treptococcus agalactiae*	288	Wellcome Trust Sanger Institute	PRJEB2837 ERP001152	(Ip et al., 2016; Sullivan et al., 2017)
*Streptococcus agalactiae* Genome sequencing	287	Baylor College of Medicine	PRJNA274384 SRP053238	(Flores et al., 2015; Teatero et al., 2016)
Persistence of a dominant bovine lineage of group B Streptococcus reveals genomic signatures of host adaptation	149	Institut Pasteur	PRJEB12926 ERP014458	(Almeida et al., 2016)
Genome diversity of spatially distinct *Streptococcus agalactiae*	116	Wellcome Trust Sanger Institute	PRJEB2589 ERP000746	(Da Cunha et al., 2014)
Genome sequences of 113 *S. agalactiae* isolates using the Illumina technology	113	Leibniz Institute of Plant Genetics and Crop Plant Research	PRJEB4456 ERP003749	(Da Cunha et al., 2014)

**Table 3:** GBS genome sequencing reports.

Strain name(s)	MLST	Serotype	Host	Location	Reference
ILRI005, ILRI112			Camel	Kenya	(Zubair et al., 2013b)
ILRI025, ILRI030, ILRI037, ILRI054, ILRI067, ILRI120, ILRI127	ST610, ST617, ST612, ST615, ST614, ST618, ST613	VI, Ia, I, V	Camel	Kenya, Somalia	(Rothen et al., 2017)
09mas018883			Cow	Sweden	(Zubair et al., 2013a)
M19		I	Cow	China	(Yang et al., 2016)
ST-1	ST1	V	Dog	United States	(Harden et al., 2016)
UCN70		III	Human	New Zealand	(Malbruny et al., 2011)
GB00112	ST17		Human	Canada	(Singh et al., 2012)
PR06			Human	Malaysia	(Mz et al., 2013)
CNCTC 10/84	ST26	V	Human	United States	(Hooven et al., 2014)
ED-NGS-1000			Human	United Kingdom	(Kropp et al., 2014)
SG-M1	ST283	III	Human	Singapore	(Mehershahi et al., 2015)
H002		III	Human	China	(Wang et al., 2015b)
GBS85147	ST103	Ia	Human	Brazil	(de Aguiar et al., 2016)
GB00037	ST1	V	Human	Canada	(Singh et al., 2016)
34 strains total	Various	Various	Human	France	(Six et al., 2016)
BSB14107, BSB14238	ST19		Human	United Kingdom	(Doumith et al., 2017)
14-179, 14-192, 13-6, 13-87, 12-165, 12-221, 12-224, 11-11, 11-19, 11-206	ST2, ST19, ST26, ST805, ST806	II, III, V	Human	Norway	(Gabrielsen et al., 2017)
874,391	ST17	III	Human	Japan	(Sullivan et al., 2017)
VB11227, VB12497, VBP4522, VBP3124	ST103, ST249, ST23, ST1	Ia, V	Human	India	(Veeraraghavan et al., 2017)
Sag37, Sag158	ST12, ST19	Ib, III	Human	China	(Zhou et al., 2017)
HU-GS5823	ST335	III	Human	Japan	(Nagaoka et al., 2018)
195-16-B-RAT, 196-16-B-RAT, 197-16-B-RAT, 198-16-B-RAT, 199-16-B-RAT, 200-16-B-RAT, 201-16-B-RAT	ST1, ST12	V, Ib	Rat	United States	(Bodi Winn et al., 2017)
STIR-CD-17	ST260	Ib	Tilapia	Honduras	(Delannoy et al., 2012)
GD201008-001	ST7	Ia	Tilapia	China	(Liu et al., 2012)
ZQ0910			Tilapia	China	(Wang et al., 2012)
SA20-06			Tilapia	Brazil	(Pereira et al., 2013)
FNA07, FPrA02, ENC06	ST7		Tilapia	Thailand	(Kayansamruaj et al., 2014)
138P			Tilapia	United States	(Pridgeon and Zhang, 2014)
138spar			Tilapia	United States	(Pridgeon et al., 2014)
JP9, JP17	ST7, ST283	Ia, III	Tilapia	Thailand	(Areechon et al., 2016)
S25	ST552	Ib	Tilapia	Brazil	(Mainardi et al., 2016)
S13	ST552		Tilapia	Brazil	(Facimoto et al., 2017)
HZAUSC001			Tilapia	China	(Zhang et al., 2017)
14-98, 14-104, 14-110	ST260, ST261	Ib	Tilapia	Honduras, Costa Rica, United States	(Jaglarz et al., 2018a)
14-66, 14-107, 14-119	ST7	Ia	Tilapia	United States, China	(Jaglarz et al., 2018b)

### Group B Streptococcus Molecular Epidemiology of Human Isolates

One of the major solutions that increases in genome sequencing throughput has delivered is definitive molecular epidemiology (Klemm and Dougan, 2016). The bacterial genomics field has variously described this also as phylogeography, phylogenomics, or global population structure studies; but the underlying concept of correlating strain relatedness with some other variable, such as isolation location, remains the same. The first major study to present enough sequences that were reasonably thought to represent nearly the full species diversity was published in 2014 (Da Cunha et al., 2014). This manuscript analyzed 229 strains, mostly (94%) isolated from humans, but also including 13 isolates from four other animals, all encompassing five different continents. This for the first time provided a global view of the species that could integrate the preceding MLST scheme. One prominent result from this analysis was the definitive conclusion that human GBS disease isolates arise from a limited number of clones. The clonal evolution and spread of individual clones was already strongly suggested by previous MLST studies, which had already identified clonal complexes that were variously associated with human and animal disease (Table 1). Interestingly, unlike several other bacterial pathogens, the distribution of GBS clones was not generally correlated with geography. There were some known exceptions [like the prevalence of CC26 in Africa (Brochet et al., 2009)], and the sampling of South America, Africa, and Asia was extremely low, both in this study and generally in the GBS literature (Dagnew et al., 2012; Johri et al., 2013; Kwatra et al., 2016). The overall conclusion from this first look, however, was that most of the major GBS CCs causing human disease had relatively low geographical stratification when compared with other surveyed bacteria.

A common theme for GBS, however, is that the general rules for the overall population are punctuated with notable exceptions. There has not been a similarly large-scale (i.e., species-wide) phylogeographic study of GBS since the Da Cunha 2014 publication. Instead, deeper analysis of individual clones, often with strong geographic biases, have added new perspectives to the distribution (and possibly evolution) of GBS in humans, and there has been encouraging recent progress in previously undersampled areas (not all using genomics) (Johri et al., 2013; Crespo-Ortiz et al., 2014; Dutra et al., 2014; Louthrenoo et al., 2014; Mitima et al., 2014; Belard et al., 2015; Bergal et al., 2015; Dangor et al., 2015; Eskandarian et al., 2015; Rivera et al., 2015; Villanueva-Uy et al., 2015; Wang et al., 2015, 2016; Le Doare et al., 2016; Seale et al., 2016, 2017; Sinha et al., 2016; Campisi et al., 2016b; Medugu et al., 2017; Slotved et al., 2017; Suhaimi et al., 2017; Veeraraghavan et al., 2017; Botelho et al., 2018; Emaneini et al., 2018; Guo et al., 2018; Li et al., 2018; Melo et al., 2018; Nkembe et al., 2018; Sigaúque et al., 2018; A’Hearn-Thomas et al., 2019; Lee et al., 2019; Mukesi et al., 2019; Nagano et al., 2019). However, as an example of the progress still needed, I could find only one study of human GBS each from Indonesia and the Philippines, and none from Vietnam, in Pubmed (Wibawan et al., 1992; Villanueva-Uy et al., 2015); Indonesia is the fourth most populous country in the world, and all three of these countries have populations that are larger than any individual European country.

There are several remarkable examples of what appears to be a single dominant clone of GBS causing the majority of disease in specific locations. From 1992 to 2013, more than 90% (210/229) of the invasive serotype V isolates were closely related isolates of an ST1 clone (however, this study did not examine other serotypes) (Flores et al., 2015). A similar serotype-restricted study found a rising incidence of serotype IV isolates in Minnesota from 2004 to 2008 [8.4% of 1,160 patients (Diedrick et al., 2010) compared to <1% of nearly 3,000 isolates from 1993 to 2002 from four cities including Minneapolis-St. Paul (Ferrieri et al., 2004)]. Subsequent genomic studies, again encompassing strains from Minnesota as well as Manitoba and Saskatchewan, Canada, determined that 89% of the serotype IV isolates from 2010 to 2014 were from the same clone of ST459 (Teatero et al., 2015a). Interestingly, in the geographically distant Toronto, where serotype V isolates were dominated by ST1, 81% of serotype IV isolates collected from 2009 to 2012 were comprised of just two STs, ST452 (CC23) and ST459 (CC1) (Teatero et al., 2015b), the same major STs previously found in Minneapolis (Diedrick et al., 2010). Additionally, there has recently been another dramatic reported expansion of a single clone causing human disease: ST283 (serotype III) in Southeast Asia (Ip et al., 2006; Kalimuddin et al., 2017) (see section “Emerging Group B Streptococcus Disease”).

### Antibiotic Resistance

The headline result from the Da Cunha et al. (2014) analysis was that resistance to tetracycline in GBS drove its increased importance for human disease (Da Cunha et al., 2014). All of the major clonal complexes infecting humans had a high (>90%) rate of tetracycline resistance, mostly mediated by the *tetM* gene. The high rate of tetracycline resistance was thought to be due to initial acquisition through a mobile genetic element (*Tn916* or *Tn5801* in all but one strain) then expansion of a subsequent clone. Importantly, the insertion position of the transposon was identical within all strains of the expanded clones causing human disease. A Bayesian analysis predicted that the divergence date of the expanded tetracycline-resistant clones corresponded well with the introduction of tetracycline for clinical use in 1948 (Da Cunha et al., 2014). This raised the possibility that the increasing virulence of GBS, or at least the rise in GBS cases, was caused by the simultaneous selection for more virulent and more tetracycline-resistant strains (Da Cunha et al., 2014).

Rising resistance is a nearly universal feature of medically important bacteria. For GBS, this has been reported not only for tetracycline, but for also for fluoroquinolones and aminoglycosides (Hays et al., 2016). Fortunately, beta-lactam antibiotics, particularly penicillins, which are first-line therapy for GBS, have remained highly effective, with large surveys documenting less than 1% of strains as resistant (Hays et al., 2016; Metcalf et al., 2017). Interestingly, vancomycin resistance has recently been reported for the first time in two GBS strains, through introduction of two different *vanG* elements found integrated at the same chromosomal locus in both strains (Srinivasan et al., 2014). Finally, macrolide resistance, most commonly measured for erythromycin, has also been rising, with rates measured since 2010 in the range of 14–59% (Lamagni et al., 2013; Da Cunha et al., 2014; Hays et al., 2016; Metcalf et al., 2017). Of great interest, a large survey in France found an exception to this trend, with rates of macrolide resistance falling from 47 to 30% between 2007 and 2014 (Hays et al., 2016). Many of these surveys leverage strong antibiotic susceptibility testing infrastructure in first-world countries; however, this is beginning to give way to genomic predictions that may eventually provide solutions for low resource settings. With regard to this, GBS-specific analyses for the prediction of antibiotic resistance and serotypes from genomic data were shown to provide high accuracy (Metcalf et al., 2017). Genomics currently cannot fully replace traditional antibiotic testing, as previously unknown (or rare) resistance mechanisms cannot be predicted from sequence data alone. However, genomics has the additional advantage of providing greater insight into the dynamics driving spread of resistance and changes in resistance rates. For example, antibiotic resistance in GBS is largely mediated by resistance gene acquisition for all of the major antibiotic classes except for fluoroquinolones, which instead arise mostly by mutations in the *gyrA* and *parC* genes and penicillins (Metcalf et al., 2017). In addition, there are multiple examples of rising resistance rates being associated with expansion of individual clones, as seen for tetracycline as described above (Da Cunha et al., 2014), erythromycin resistance in ST1 (Flores et al., 2015), and beta-lactam resistance in several examples of closely related isolates (Metcalf et al., 2017). Overall, therefore, genomics paints a general overall picture similar to that described for clonal emergence above; antibiotic resistance is initially acquired through horizontal transfer or possibly recombination (also enabling acquisition of fluoroquinolone or penicillin resistance), followed by clonal expansion of successful lineages that drive increases in antibiotic resistance rates.

As in other bacteria, mobile genetic elements are often associated with more than just antibiotic resistance genes. Many of these additional genes have features that suggest they may be involved in virulence, such as surface attachment signals (LPXTG), predicted surface localization, homology to adhesin proteins, novel metabolic activities, or predicted secretion and toxicity. One well-described example is the co-occurrence of AlpST-1, a predicted surface-exposed adhesin protein, that is encoded within the same mobile genetic element (denoted the RDF.2 MGE) as the *tetM*-carrying *Tn916* in a collection of 202 ST1, serotype V strains from the US and Canada (Flores et al., 2015). In this clone, the authors argue that the close genetic linkage between *tetM* and the AlpST-1 virulence gene could account for the association between tetracycline resistance and virulence, so the virulence would not be due to the tetracycline resistance *per se* (Flores et al., 2015).

The concept that antibiotic resistance is associated with strains with high pathogenic potential for humans (and other animals) is not controversial. Indeed, therapeutic and agricultural antibiotic usage is perhaps one of the strongest influences that humans have exerted on the makeup of our microbial environment. However, we typically associate antibiotic resistance with a loss of fitness in bacteria, which is then overcome by the strong selection pressure of antibiotic administration (though numerous counterexamples exist). Antibiotic resistance is generally not considered a virulence determinant in and of itself. The observation of high tetracycline resistance rates, independently acquired in multiple lineages of common human GBS strains (CC1, 10, 17, 19, 23, and 26), is indeed an incontrovertibly strong demonstration that tetracycline use has dominated the evolution of GBS in humans (Da Cunha et al., 2014). As in the case of ST1, however, the underlying pathogenic mechanisms, which can lead to a stronger understanding of disease and novel strategies for prevention (Flores et al., 2015), remain the central unanswered question for essentially all GBS lineages.

### Group B Streptococcus Molecular Epidemiology in Cattle

In contrast to the idea that most of the GBS population was not strongly stratified by geography, it has long been known that GBS demonstrates relatively strong host specificity. In addition to human disease, the economic impact of lower milk production due to mastitis has driven substantial *S. agalactiae* research (Keefe, 1997; Ruegg, 2017). Substantial work, therefore, has also examined the potential commonality in the rise of human and cattle infections, most obviously mediated through milk and close contact with dairy farmers (Bliss et al., 2002; Bohnsack et al., 2004; Sukhnanand et al., 2005; Foxman et al., 2007; Manning et al., 2010).

The literature describing GBS that infects bovine hosts is extensive, in keeping with the initial *S. agalactiae* nomenclature (which refers to its effects on dairy cows). As with human isolates, pre-genomic techniques had already been used to sketch a general outline of the population structure. Bovine isolates generally fall into two main clusters, represented in the MLST scheme by CC67 and CC23. A significant minority of strains were found to fall into CCs that overlapped with human isolates, most prominently CC17 (Sørensen et al., 2010). As seen with human GBS, however, there are examples of recent changes in prevalent GBS clones which may differ based on geography. A recent rise in CC61 strains was noted in Portugal, with an origin estimated in the early 1990s (Almeida et al., 2016). From 84 bovine isolates collected from milk from 14 dairy farms in China between 2011 and 2016, all were either CC61 or CC103 (Pang et al., 2017). Interestingly, CC103 strains have also been noted in Denmark and Norway, but not the UK or US (Bisharat et al., 2004; Zadoks et al., 2011; Jørgensen et al., 2016).

The fact that GBS was known first as a bovine pathogen and only later noted to cause increasing rates of neonatal and adult disease led to a strong interest in exploring a potential zoonotic transmission from cows to humans (and *vice versa*). There was suggestive increase in GBS colonization rate among university students who drank milk (*n* = 150), but this was not statistically significant (Bliss et al., 2002). A larger, longitudinal study (3-week intervals over 3 months) in a similar population found no significant association between GBS colonization and beef or milk consumption; notably, there were positive associations with sexual activity and fish consumption (Foxman et al., 2007). Another epidemiological study examined the colonizing GBS in humans who had regular close contact with cattle. Of 68 matched human-cattle stool samples, one set had the same GBS as measured by MLST, capsule, RAPD pattern, and antibiotic resistance profile. Of note, the cattle sampled were not symptomatic, and stool is not a typical sampling location in cattle. Perhaps more interestingly, human colonization was significantly associated with exposure to cattle in the previous week (Manning et al., 2010). Overall, therefore, transmission between humans and cattle seemed rare if not nonexistent.

The conclusion that the human pathogenic CC17 lineage was derived from a bovine GBS ancestor was therefore a dramatic result (Bisharat et al., 2004). This initial report was based on an analysis of MLST gene sequences. In what has become a repeated testament to the value of whole genome analyses, this initial MLST result has been challenged by subsequent genomic studies (Brochet et al., 2006; Sørensen et al., 2010; Richards et al., 2013). Comparative whole genome hybridization on microarrays to assess gene content in a collection of 75 strains from humans and multiple animal hosts indicated that the common ancestor of the bovine ST61 and ST17 strains was likely more similar to a human ST17 strain, implying the reverse transmission direction (Brochet et al., 2006). An examination of 15 genes (including the seven MLST genes) in addition to several virulence-associated traits in a representative set of 55 strains (drawn from 238 in total) showed differences between the human and bovine CC17 strains that were not captured by the MLST genes. Combined with differences between human and bovine strains with respect to the ability to ferment lactose, the presence of the human-associated *scpB* virulence gene, and the presence of the PI-1 pilus island, the authors concluded that human CC17 strains evolved separately, perhaps from a common diverse pool, from the bovine CC17 isolates (Sørensen et al., 2010). Finally, whole genome sequencing data from 202 strains isolated from human and animal hosts was used as part of an analysis aimed at understanding the factors responsible for survival in the bovine mammary gland. Acquisition of the Lac.2 operon, which enables fermentation of lactose (the primary sugar in cow milk), was a consistent feature of bovine strains. In contrast, among ST17 strains of human and bovine origin, none of the human strains had the Lac.2 operon, which was interpreted as unlikely if a bovine strain was the ancestor of the human isolates (Richards et al., 2013). Notably, as with the association of human strains with presence of the *scpA* and *lmb* genes noted above, the association of the Lac.2 operon with bovine isolates, while strong, is not exclusive; 8/151 human strains in one survey carried Lac.2 (Richards et al., 2013), though for some strains, such as NEM316, the origin (possibly *S. gordonii*) may be distinct from the origin suspected in bovine strains (*S. dysgalactiae* subsp. *dysgalactiae*) (Richards et al., 2011). Therefore, with the benefit of larger scale genomic data, it still appears that bovine and human strains are largely (if not completely) separate, at least in terms of the severe disease-causing strains, though the possibility of overlap continues to be discussed (Lyhs et al., 2016; Wang et al., 2018b). There seem to be cases of colonization in both directions, but this appears to be transient in both cattle and humans (Jensen, 1982; Betsy Foxman et al., 2006). On a longer evolutionary scale, however, the genomic data are clear that some bovine- and human-adapted lineages do at least share common ancestors, most notably for CC17 and CC67 (Sørensen et al., 2010; Pang et al., 2017).

### Group B Streptococcus Molecular Epidemiology in Fish

The next most extensively studied host organism for GBS is fish. Again, much of the motivation for this research has been economic interest, as Streptococcosis is a major disease affecting farmed fish (Amal et al., 2011). In fish, GBS are generally limited to certain MLST types, which leads to their occasional reference in this literature as biotypes, with a strong association with serotype. One early study proposed two new species, *S. shiloi* and *S. difficile*, as pathogens of fish in Israel starting in 1986 (Eldar et al., 1994). These were later resolved to *S. iniae* and *S. agalactiae* (serotype Ib), respectively (Eldar et al., 1995; Vandamme et al., 1997). Subsequently, the fish literature began to refer to biotypes of GBS, which persists today in the marketing for fish vaccines^1^. Biotype I corresponds to serotype Ia, ST7, and is mostly found in Asia. Biotype II corresponds with serotype Ib, ST260 or ST261 (and is referred to as CC552, though this has recently been refined), and is found generally throughout the globe (Delannoy et al., 2013; Paul, 2014; Munang’andu et al., 2016; Barony et al., 2017).

Similar to the situation with bovine GBS, one major topic of research has been the possibility of cross-species infections between humans and fish. Reliance on MLST again indicated that there was evidence for transmission: ST7 is found in both humans and fish (Evans et al., 2008; Delannoy et al., 2013), as well as other aquatic animals, and a large outbreak of fish infections in Kuwait Bay was thought to be due to human GBS isolates entering the water through sewage contamination (Jafar et al., 2008). Three genomic studies later examined human and fish ST7 strains more closely. In one study, the human and fish ST7 strains were very similar, with similar gene content and a uniformly low nucleotide divergence throughout the genome (Rosinski-Chupin et al., 2013). A second study noted that human and piscine ST7 strains were closely related based on genome content (CRISPR arrays, prophages, and virulence-associated genes) (Liu et al., 2013). The third study, which included fish strains from both CC7 and CC552, identified eight genomic regions, mostly located within genomic islands, containing genes associated with or specific to piscine strains, which were then confirmed by PCR screening across a larger collection of 43 isolates from various hosts (Delannoy et al., 2016). In addition, strains of ST23, which are common human pathogens, were unable to cause experimental infections in tilapia, while one of two human ST1 strains was equally pathogenic to tilapia compared a *bona fide* piscine ST7 isolate (Delannoy et al., 2016; Wang et al., 2017). Overall, therefore, it appears that much of the population of piscine and human isolates are largely separate, but at least some strains of CC7, may be able to infect both hosts. Another common theme is the idea that host specificity has evolved through genome reduction, where genes and pathways no longer needed in other environments (hosts such as humans) are lost once GBS specializes to colonize fish, most clearly demonstrated in CC7 and CC552 (Liu et al., 2013; Rosinski-Chupin et al., 2013; Delannoy et al., 2016). For example, several studies found multiple fish strains with genome sizes in the 1.7–1.8 Mbp range, compared with 2.0–2.2 Mbp for many human isolates (Liu et al., 2013; Rosinski-Chupin et al., 2013). This 200–300 Kbp accounts for over 100 otherwise “core” genes lost in fish isolates, which are enriched for carbohydrate transport and metabolism functions (Liu et al., 2013). Interestingly, another study of human ST19 strains found that at least some are able to cause experimental disease in tilapia, a phenotype that correlates with capsule type (Wang et al., 2018a).

One of the notable features of the research on piscine GBS is that the sampling covers a complementary geographic range to the human and much of the cattle GBS literature. Aquaculture is a rapidly growing business in South America, the Middle East, and Asia, particularly Southeast Asia; these are all areas that have been notably undersampled in human-focused GBS studies. In these areas, it appears that many of the major food fishes, found in both freshwater and saltwater, can be affected (including rainbow trout, seabream, tilapia, yellowtail, catfish, croaker, killfish, and pomfret) (Amal et al., 2011). Interestingly, our knowledge of the population structure of piscine GBS is actually stronger than that for human disease-causing isolates in many countries, for a variety of reasons including economics, infrastructure, and the assumption that seemingly globally distributed clones (for human disease) would be similarly dominant in unsampled regions. Accordingly, there are potentially emerging clones of piscine GBS that are being reported (such as Serotype III and IX in Southeast Asia and China, respectively) (Kalimuddin et al., 2017; Zhang et al., 2018).

### Emerging Group B Streptococcus Disease

The emerging GBS clone in Southeast Asia, ST283 (Serotype III), neatly intertwines the previously mentioned themes of ongoing GBS evolution (leading to potential cross-species host jumps), the potential for some clones to have strong geographical associations, and the value of genomics for enabling integration across different disciplines. Emerging clones such as ST283 therefore challenge our understanding of GBS ecology, evolution, disease, and management.

In 2015, Singapore experienced an outbreak of foodborne GBS infections. Associated with consumption of a local raw fish dish (魚生, yu sheng), over 200 patients suffered severe invasive disease, with bacteremia, meningitis, and septic arthritis (Rajendram et al., 2016; Tan et al., 2016; Kalimuddin et al., 2017)^2^. Prior to this outbreak, GBS had never been thought to be transmitted by the foodborne route. In retrospect, there had been indications that food consumption was associated with gastrointestinal colonization (Bliss et al., 2002; Foxman et al., 2007), and there had been an example of lizards contracting GBS sepsis through consuming contaminated mice (Hetzel et al., 2003). Furthermore, it seems clear that GI colonization by any organism in humans seems reasonable to assign to oral consumption until proven otherwise. Given that GBS is a common GI colonizer of both men and women, then, one interpretation is that even early onset neonatal meningitis is ultimately a foodborne disease (*via* vaginal colonization through the GI tract, then infecting the newborn during birth).

Interestingly, the outbreak organism, ST283, is a common MLST type that causes aquaculture-associated fish Streptococcosis. An ST283 strain and a single locus variant, ST491, had been identified in farmed fish in Vietnam and Thailand, isolated in the early 2000s (Delannoy et al., 2013). ST283 strains had also been reported to cause invasive infections similar to those seen in the 2015 Singapore outbreak in otherwise healthy humans from Hong Kong and Singapore as early as 1998 (Wilder-Smith et al., 2000; Ip et al., 2006, 2016; Barkham et al., 2018). Investigating after the 2015 Singapore outbreak, it was found that 71% of freshwater fish sold for raw consumption (which was associated with the outbreak) in Singapore carried GBS, with 14% carrying ST283; in contrast, 9–33% of saltwater fish carried GBS, with none being ST283 (Chau et al., 2017). Singapore imports the vast majority (>90%) of its food, including fish^3^; indeed, 4.6% of fish samples were already positive for GBS, with 1% of fish positive for ST283, at entry ports to Singapore (Chau et al., 2017), leading to a suspicion that regional aquaculture fish may also be colonized by ST283 (while ST283 is known to cause disease outbreaks in farmed fish, the fish being imported and sold appeared healthy). Troublingly, a recent report has identified ST283 as a cause of GBS outbreaks in at least five fish farms in 2016–2017 from four different states in Brazil, and this is suspected to be due to import of fish from Southeast Asia (Leal et al., 2019).

ST283 was originally described an emerging cause of invasive infections in Hong Kong and Singapore (Ip et al., 2006). It was only identified as a potential foodborne pathogen in 2015 (Rajendram et al., 2016; Tan et al., 2016; Kalimuddin et al., 2017). Epidemiological data for human infections are not consistently available in many countries in Southeast Asia, but recent data suggest that the endemic incidence of ST283 infections outside Singapore may be comparable to or higher than the incidence at the peak of the Singapore outbreak (Barkham et al., 2019; https://outbreakwatch.blogspot.com/2018/07/proahedr-strep-group-b-singapore.html). If ST283 is also a foodborne pathogen outside Singapore, as recently suspected in Thailand (Kayansamruaj et al., 2018), this suggests the remarkable interpretation that GBS infections in Southeast Asia are actually first and foremost a foodborne illness (in stark contrast to the current paradigm of neonatal and immunocompromised disease found in current medical teaching). This further raises the possibility that invasive GBS disease caused by non-ST283 strains in human adults may be at least partially foodborne, expanding our understanding of GBS in general as a possibly long unappreciated zoonotic pathogen, with attendant implications for food safety. As food consumption is associated with vaginal colonization in women (Foxman et al., 2007), a further implication is that neonatal infections may also ultimately be a late sequelae of foodborne consumption of GBS. Interestingly, there is a case report of a late-onset neonatal infection caused by a genomically indistinguishable strain that was consumed by the mother *via* placental pills (Buser et al., 2017).

## Conclusions and Outlook

GBS has a uniquely dynamic biology. At the overall species level, there is remarkably broad host range and global geographical reach. Standing in counterpoint to this broad generalism are numerous examples of strong host specificity and local geographical stratification. The source of this intraspecies heterogeneity likely traces back to similarly dynamic processes shaping the genome, with the capacity for large-scale chromosomal recombinations paired with highly independent, clonal evolution of individual successful lineages. GBS is not only an emerging pathogen in the traditional sense of rising incidence; a closer examination of its biology indicates that it is a continuously emerging pathogen that, in little more than a single human lifetime, has altered and continues to alter the tenets of epidemiology of human, bovine, and piscine colonization, infection, and economics. Since the first GBS genome sequences, genomics has been an ideal technology to capture the dynamism of this species. It therefore seems apt that GBS was the organism that birthed the concept of the pan-genome; its description as having an open pan-genome, with infinite possibility for evolution and adaptation, is a fitting metaphor for the recent history of GBS. GBS, in turn, provides a compelling argument for the continued progress of genomics and our need, as a community, to implement broad genomic monitoring in both developed and developing countries. The recent genomic history has demonstrated shifts in our understanding of GBS with respect to cattle, human neonates, human adults, aquacultured fish, and now the interface between fish and humans and food safety and economic development. Where else is GBS lurking? Further advances in genomics will hopefully enable us not only to reconstruct *post hoc* what GBS will do next, but to catch it in the early stages of its next evolutionary jump.

## Author Contributions

The author confirms being the sole contributor of this work and has approved it for publication.

### Conflict of Interest Statement

SC is a co-inventor on a patent application for a GBS ST283-specific test.

## Footnotes

^1^https://www.aquavac-vaccines.com/products/

^2^http://10minus6cosm.tumblr.com/post/132590510556/serious-group-b-streptococcal-infection-in-adults

^3^https://www.ava.gov.sg/
